# Electron energy-loss spectroscopy of branched gap plasmon resonators

**DOI:** 10.1038/ncomms13790

**Published:** 2016-12-16

**Authors:** Søren Raza, Majid Esfandyarpour, Ai Leen Koh, N. Asger Mortensen, Mark L. Brongersma, Sergey I. Bozhevolnyi

**Affiliations:** 1 Centre for Nano Optics, University of Southern Denmark, Campusvej 55, DK-5230 Odense M, Denmark; 2 Geballe Laboratory for Advanced Materials, Stanford University, 476 Lomita Mall, Stanford, California 94305, USA; 3 Stanford Nano Shared Facilities, Stanford University, Stanford, California 94305, USA; 4 Department of Photonics Engineering, Technical University of Denmark, DK-2800 Kgs. Lyngby, Denmark; 5 Center for Nanostructured Graphene (CNG), Technical University of Denmark, DK-2800 Kgs. Lyngby, Denmark

## Abstract

The miniaturization of integrated optical circuits below the diffraction limit for high-speed manipulation of information is one of the cornerstones in plasmonics research. By coupling to surface plasmons supported on nanostructured metallic surfaces, light can be confined to the nanoscale, enabling the potential interface to electronic circuits. In particular, gap surface plasmons propagating in an air gap sandwiched between metal layers have shown extraordinary mode confinement with significant propagation length. In this work, we unveil the optical properties of gap surface plasmons in silver nanoslot structures with widths of only 25 nm. We fabricate linear, branched and cross-shaped nanoslot waveguide components, which all support resonances due to interference of counter-propagating gap plasmons. By exploiting the superior spatial resolution of a scanning transmission electron microscope combined with electron energy-loss spectroscopy, we experimentally show the propagation, bending and splitting of slot gap plasmons.

## Abstract (web summary)

Here Raza *et al*. use using scanning transmission electron microscopy combined with electron energy-loss spectroscopy to provide detailed characterization of gap surface plasmon modes supported by a freely suspended silver nanoslit of 25 nm width.

Manipulating the flow of electromagnetic waves on the nanoscale has been envisioned as the solution to interface nanometre-sized electronic circuits with diffraction-limited optical waveguides. For nanoscale control, the most promising approach is to couple propagating electromagnetic waves in dielectric media, such as optical fibres, to surface-plasmon waves supported on nanostructured metallic surfaces^1^,^2^,^3^. Surface plasmons are collective oscillations of the free electrons, and being confined to the metal surface they have the ability to localize light greatly beyond the diffraction limit and down to dimensions that bridge optoelectronics to electronic integrated circuits^4^. A plethora of surface-plasmon modes exists, which may be divided into two overall classes: localized surface-plasmon resonances in confined nanoparticles and propagating surface plasmons in extended waveguides. Given its propagating nature, the latter is an ideal candidate for light manipulation on the nanoscale in integrated optical nanocircuits that can perform functions such as high-speed processing, routing or modulation^5^,^6^.

While there are many different types of propagating surface plasmons^4^, including nanoparticle chains^7^, long-range surface plasmons^8^, graphene plasmons^9^ and hybrid plasmonic–photonic modes^10^, it is of paramount importance for nanocircuitry applications that the information-carrying plasmonic mode has strong mode confinement with significant propagation length. In addition, properties such as single-mode operation and broadband guiding are needed. Despite the fundamental plasmonic trade-off between mode confinement and propagation length^11^, gap surface plasmons (GSPs), which propagate in a dielectric medium or air gap between metal surfaces^12^,^13^, provide the required strong mode localization and micrometre propagation lengths^14^, making them suitable for integrated optics. Guiding of GSPs has been experimentally realized in several different geometries^15^, such as V-grooves^16^, slot waveguides^17^,^18^,^19^,^20^,^21^ and metal–insulator–metal (MIM) waveguides^22^. So far, one of the most promising GSP-based waveguides for subwavelength circuitry has been the slot waveguide^5^,^6^,^18^,^19^. Theoretical investigations^23^,^24^,^25^,^26^ of narrow slot waveguides have provided strong evidence for the aforementioned desired modal properties of the slot GSP, as well as an expected tolerance to fabrication imperfections and high transmittance through sharp bends^27^. The slot GSP is therefore theoretically expected to be an ideal candidate for optical nanocircuitry, which we support experimentally in this work.

Characterizing the modal properties of the extremely confined GSP mode in nanosized slot waveguides using optical techniques, including even near-field measurements^20^, is a next to impossible task due to limited spatial resolution. In contrast, electron energy-loss spectroscopy (EELS) performed in a scanning transmission electron microscope (STEM) can probe plasmonic response on the nanoscale^28^,^29^. STEM EELS is a powerful characterization tool due to the combination of Angstrom spatial resolution with millielectronvolt spectral resolution over a broad energy range^30^,^31^. In recent years, STEM EELS has proved to be an indispensable tool for optical characterization^28^ and has been utilized in many diverse plasmonic studies^32^,^33^,^34^,^35^,^36^,^37^,^38^,^39^,^40^,^41^,^42^,^43^,^44^.

In this work, we use STEM-EELS to characterize the GSP mode supported by freely suspended silver (on silicon nitride) slot waveguide resonators of only 25 nm width, which is several times narrower than state-of-the-art slot waveguides^6^,^18^,^19^. We study both straight and branched slot waveguide resonators for nanocircuitry applications. Besides providing experimental evidence for the important broadband propagation properties of the slot GSP, we also show that 90° bending with negligible back reflection can be achieved, which is required for nanoscale light routing. Light modulation often requires the splitting and interfering of several beams, which in both cases occurs at junctions in the optical circuit. By examining cross- and T-shaped junctions in the slot waveguide resonators, we show that splitting can also be achieved with the slot GSP. On another note, the spectral positions of the GSP-induced excitations of the slot resonator depend strongly on the optical path length, which can be exploited for refractive index sensing. In fact, we show that the sensitivity of our nanoscale resonator is comparable to state-of-the-art plasmonic sensors of similar footprints^45^. The ease of fabrication and the desirable optical properties make the slot GSP of both fundamental and practical interest with a wide variety of plasmonic applications.

## Results

### Sample preparation

**Figure 1:**
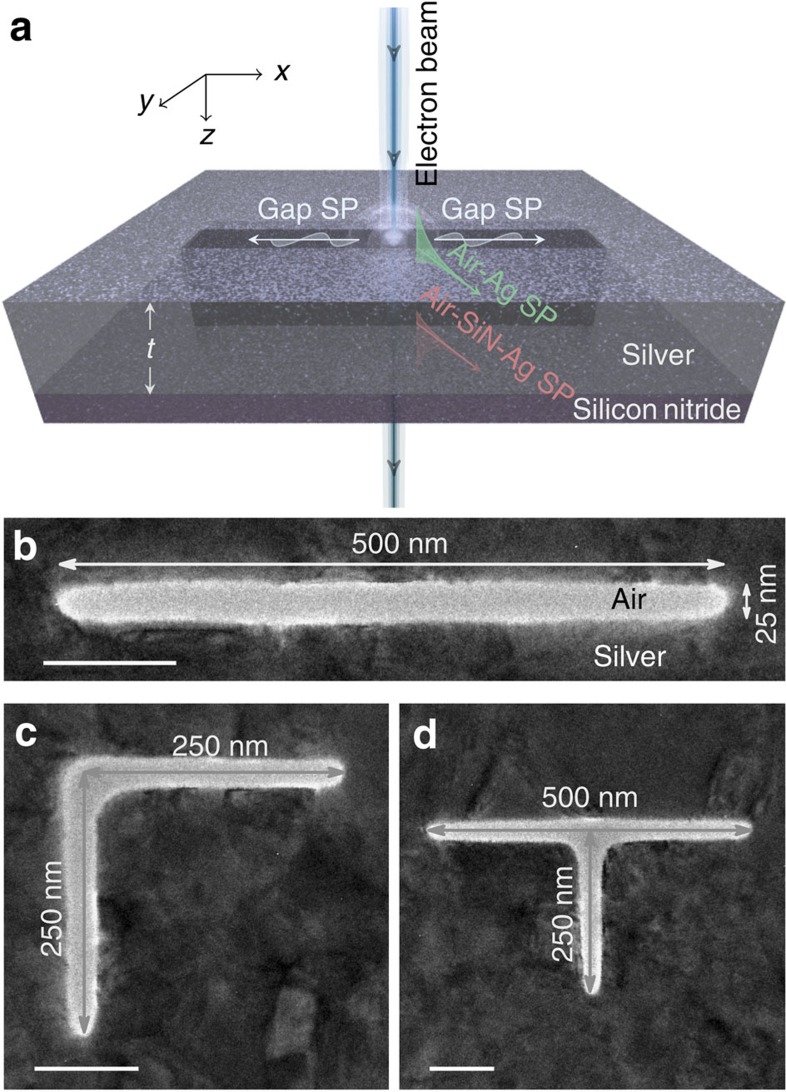
Gap surface-plasmon resonators. (**a**) Artistic impression of a swift electron beam interacting with a straight slot of thickness *t*. The electron beam excites gap surface plasmons inside the slot and regular surface plasmons at the top (silver-air) and bottom (silver–silicon nitride-air) interfaces. (**b**–**d**) Plan-view TEM images with size labels of some of the fabricated slot geometries, for example, (**b**) straight, (**c**) L shape and (**d**) T shape nanoresonators. Scale bar, 100 nm.

The samples are prepared by first depositing a silver film on a 10 nm thick silicon nitride transmission electron microscope (TEM) membrane. Slot geometries of different shape are subsequently fabricated by milling both the silver film and the silicon nitride substrate using a focused ion beam (FIB), see Fig. 1a for a schematic illustration of a straight slot waveguide resonator. As the lateral confinement (that is, in the *y* direction) of light is crucial for miniaturization of integrated optical circuits, the width of the slots should be as narrow as possible, that is, at the resolution limit of the FIB. The lateral spatial resolution of the FIB procedure and the ability to mill vertical side walls in the slot degrades with increasing silver film thickness *t*, making the deposition of a very thin silver film attractive. However, we must simultaneously ensure that the GSP supported by the slot should have the same favourable properties as that of the MIM GSP (corresponding to an infinitely thick silver film), such as strong mode localization, significant propagation length in a broad spectral range and single-mode operation. To this end, we have numerically verified that the effective index of the slot GSP mode for a thickness of *t*=150 nm (Supplementary Fig. 1) provides the desired modal properties along with ease of fabrication, which is why this thickness of the silver layer is chosen for the experimental study.

To study the guiding, bending and splitting of light on the nanoscale, we choose to fabricate straight, L-shaped and T-shaped slot resonators, respectively. Plan-view TEM images of selected fabricated structures can be seen in Fig. 1b–d. Here we see that all of the slots are extremely narrow with widths *w* of ∼25 nm, a size limited by the resolution of the FIB procedure. Expectedly, the width decreases in vicinity of the slot terminations, while at corners and junctions it becomes slightly larger. For the straight and L-shaped slot resonators (Fig. 1b,c), a total resonator length of ∼500 nm is chosen. While the branches of the L-shaped slot resonator in Fig. 1b are each ∼250 nm, we have also fabricated other L-shaped resonators with different branch sizes while keeping the total resonator length fixed at ∼500 nm. Finally, the T-shaped slot resonator shown in Fig. 1d has an upper branch of length 500 nm and a lower branch of ∼250 nm. T-shaped resonators with shorter lower branch lengths (approximately 125 and 170 nm) have also been fabricated and characterized. Importantly, as all of the prepared slot structures are on the same TEM membrane, the EELS characterization of all resonators can be carried out in the same microscope session (see Methods section), thereby eliminating any uncertainties associated with realignment between measurements of different resonators.

### Straight slot

**Figure 2:**
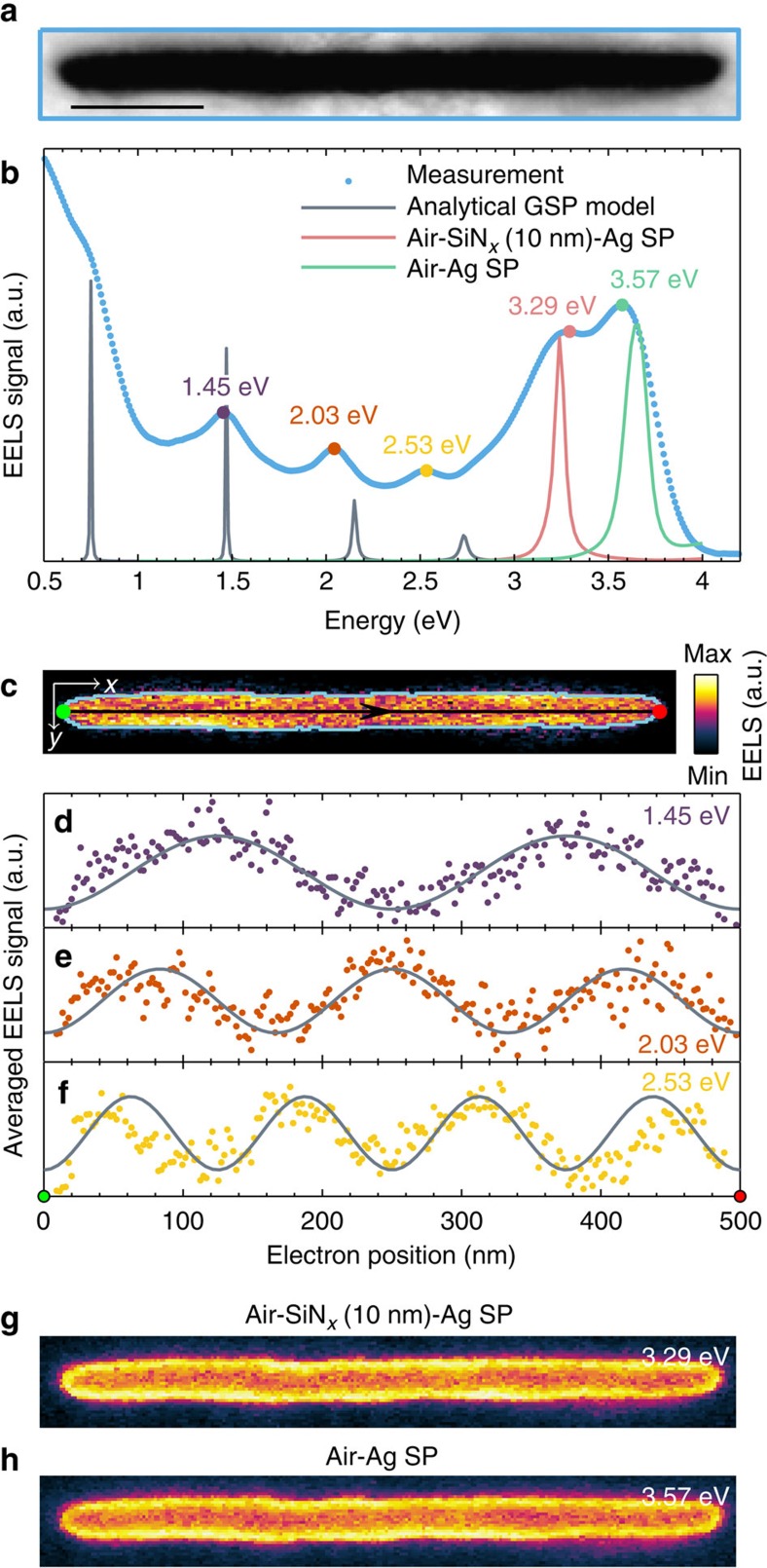
Straight slot waveguide resonator. (**a**) STEM image of straight slot waveguide resonator with length *L*≈500 nm and width *w*≈25 nm. Scale bar, 100 nm. (**b**) Total EELS signal from the enclosed blue box in **a** along with theoretical calculations using the analytical GSP model and simulations of electron-excited surface plasmons. (**c**) 2D EELS intensity map from a spectral window of 0.15 eV centred at the resonance energy 1.45 eV. The light-blue line indicates the boundary of the air gap, which is determined from the STEM image in **a**. (**d**) Transversally averaged EELS signal of the map in **c** within the boundary of the air gap along with calculations using the analytical GSP model. (**e**,**f**) Similar to **d** but at the resonance energies 2.03 and 2.53 eV, respectively. (**g**,**h**) 2D EELS intensity maps at the surface-plasmon resonance energies 3.29 and 3.57 eV, respectively, showing uniform EELS distribution along the air–silver boundary.

We begin by studying the subwavelength plasmon guiding properties of the slot by considering a straight slot waveguide resonator of length *L*≈500 nm and width *w*≈25 nm, see Fig. 2a for the STEM image. To map all of the plasmon modes supported by the slot, we record EELS data over a rectangular grid covering the entire slot (blue line in Fig. 2a) with a pixel size of ∼2 nm (see Methods for details). With this technique, usually referred to as spectrum imaging^32^, we end up with a data cube with two spatial indices and one energy index. By summing over the two spatial indices, we end up with a single position-independent EELS spectrum, shown in Fig. 2b, which contains loss events from all of the plasmon modes of the slot. Figure 2b displays five distinct resonances, where the three lower-energy resonances are less pronounced than the two higher-energy resonances at 3.29 and 3.57 eV, indicating that the plasmon modes producing the latter resonances are more easily coupled to by the electron beam. Due to the positive-energy tail of the immense zero-loss peak^46^, the resonance at ∼0.75 eV is visible only as a weak shoulder. For further insight into the mode characteristics of each resonance, we visualize the spatial distribution of the EELS signal in a spectral window of 0.15 eV energy width centred at the resonance energy. Such an EELS intensity map for the resonance energy *E*=1.45 eV is shown in Fig. 2c, where an increased EELS signal is observed at specific positions in the air gap (boundary shown as a light-blue line). Interestingly, the EELS pattern is harmonic along the *x* direction near the upper and lower air–silver interfaces, indicating that the excited plasmon mode resides in the gap and propagates along the *x* direction. Such harmonic EELS patterns have also previously been observed in the study of complementary structures, which illustrate Babinet's principle^47^,^48^,^49^. To highlight the plasmon propagation, we average the EELS signal in the air gap transverse to the propagation direction (that is, in the *y* direction), producing the one-dimensional EELS line profile in Fig. 2d, which shows a clear harmonic pattern with two maxima. Proceeding in an identical manner with the EELS intensity maps for the resonance energies 2.03 and 2.53 eV produces the harmonic line profiles with three and four maxima in Fig. 2e,f, respectively. We interpret the harmonic patterns of Fig. 2d–f as standing-wave resonances due to counter-propagating slot GSPs in the resonator (see also Supplementary Fig. 2 for the dispersion relation), although we note that the EELS pattern cannot directly be interpreted as the electric field profile^50^. Instead, as we will see from later theoretical considerations, the EELS pattern can be understood as the GSP excitation efficiency of the electron. Finally, we consider in Fig. 2g,h the EELS intensity maps for the resonance energies 3.29 and 3.57 eV, respectively. Here we note that the two EELS intensity maps are quite similar to each other with a strong EELS signal near the air–silver boundary, but different from the harmonic pattern observed in the lower-energy resonances. This suggests that the plasmon modes of these two resonances are similar in nature, but distinct from the GSP-induced resonances. Indeed, this difference in mode characteristic is emphasized when averaging the EELS intensity maps along the longitudinal direction (that is, *x* direction), where it becomes evident that the EELS signal of the GSP-induced resonances is maximized inside the air gap several nanometres from the air–silver boundary, while the maximum EELS signal of the high-energy plasmon modes is observed exactly at the air–silver boundary (Supplementary Fig. 3). By calculating the EELS response from electron-excited surface plasmons at planar silver–air and silver–silicon nitride–air interfaces in the non-retarded limit (Methods), we find the EELS signal shown in green and red lines in Fig. 2b, respectively. A single resonance appears in each case, which match in energy with our observed high-energy resonances. Thus, we interpret these two resonances as due to the coupling of the electron beam to the usual surface plasmons at the top (silver–air) and bottom (silver–silicon nitride–air) interfaces of our sample, as schematically illustrated in Fig. 1a. The significant thickness of the silver film (∼150 nm) gives rise to negligible hybridization of the planar surface plasmons, allowing us to model them individually. As the *z* component of the electric field of the planar surface plasmons is considerably stronger than that of the slot GSP, this interpretation also explains the much larger EELS signal observed from the surface-plasmon modes compared to the slot GSPs. As our interest is only in the slot GSP, we continue with a focus on the EELS data below 3 eV.

To understand the experimental EELS data of the GSP-induced resonances, we complement the experimental results with theoretical considerations. Although there exists several approaches to calculating the EELS signal by solving the retarded Maxwell's equations in a particular geometry^37^,^40^,^51^,^52^,^53^,^54^, we are primarily interested in the physical mechanism of the resonances and therefore adopt a simpler, yet accurate approach to determining the EELS signal. We model the resonator as a one-dimensional slit of length *L* surrounded by silver and with effective index *n*_eff_(*w*), which depends on the slit width *w*. The electron beam is for simplicity assumed to act as a point source, which generates forward- and backward-propagating GSP waves described as plane waves with wave number *k*=(*ω*/*c*)*n*_eff_ (Supplementary Note 1; Supplementary Fig. 4). By tracking the propagation and reflection of the GSP waves in the slot, we calculate the induced electric field in the slot and, hence, the EELS signal Γ(*x*_0_, *ω*) as (Supplementary Note 1)




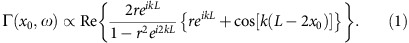




In equation (1), *r* denotes the complex reflection coefficient at the slot-silver terminations and *x*_0_ is the electron position in the slot (0<*x*_0_<*L*). Thus, with a simple one-dimensional model of the slot accounting for the counter-propagating GSP waves we arrive at equation (1), which describes both the position- and frequency-dependent EELS signal associated with Fabry–Perot resonances in the finite-length slot. To compare with our experimental EELS data in Fig. 2b, we also calculate the position-averaged EELS signal Γ(*ω*) as




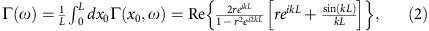




where we see that the resonance condition for the GSP-induced modes is 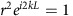. In fact, by rewriting this relation as 2*kL*+2arg(*r*)=2*mπ*, where arg(*r*) is the phase accumulation upon reflection and *m* is a positive integer, it becomes clear that resonance occurs when the accumulated round-trip phase equals an integer value of 2*π*, like it is the case for any other Fabry–Perot problem. Although the width of the slot enters through the wave number *k*, the main geometric parameter determining the resonance condition is the resonator length *L*. In addition, we note that the parameter *m* describes the mode number by relating to the number of maxima in the standing-wave pattern of the EELS signal, meaning that, for example, the *m*=1 mode corresponds to a standing-wave pattern with one maximum.

To utilize equations (1) and (2), we approximate the reflection coefficient with the Fresnel reflection for a normally-incident plane wave




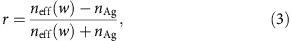




where 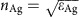 is the complex refractive index of silver taken from literature^55^. Finally, the GSP effective index is approximated by the analytical relation for the GSP mode of the metal-insulator-metal (that is, infinite thickness *t*→∞) geometry^14^,^15^




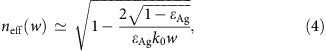




which is valid for *w*>2/(*k*_0_|*ɛ*_Ag_|), appropriate for our structures. By comparison with numerical simulations of the effective index of the GSP mode of the finite-thickness (*t*=150 nm) slot, we find that equation (4) accurately describes the effective index up to an energy of ∼2.5 eV (Supplementary Note 1; Supplementary Fig. 1). For energies above 2.5 eV, both the real and imaginary parts of the effective index of the finite-thickness slot GSP mode increase markedly and can no longer be described by the simple relation in equation (4). Notably, most of the experimentally observed resonances occur below this energy, justifying our use of the analytical MIM relation to approximate the GSP mode of the slot. Hence, with equations (3) and (4) we can now analytically calculate the GSP-induced EELS spectrum and EELS intensity maps by using equations (2) and (1), respectively.

Figure 2b compares the theoretical EELS spectrum (equation (2)) with the experimental data, where we see that the analytical GSP model accurately captures the GSP-induced resonances observed experimentally. Impressively, the resonance energies for the two first GSP modes match almost exactly with measurements, although the first-order GSP mode (*m*=1) is only weakly observed as a shoulder at approximately *E*=0.75 eV. For increasing resonance energies (that is, *m*=3 and *m*=4), the model predicts slightly larger energies than observed experimentally, which is to be anticipated from the approximative nature of equation (4). Besides the resonance energies of the GSP-induced modes, the analytical GSP model also displays a decreasing resonance amplitude and increasing full-width at half-maximum with increasing mode number. Both features are in good agreement with experimental observations, except for the mode *m*=1, which is masked due to the strong background EELS signal at low energies. Finally, we note that the experimentally measured resonances appear spectrally broader than predicted by our analytical model. We attribute this difference in linewidth primarily to the limited energy resolution of EELS (0.10 eV) along with an increased imaginary part of the permittivity in silver due to gallium ion implantation from the FIB milling.

In addition to the spectral behaviour of the EELS signal, we also compare the spatial modal distribution of our analytical GSP model with the transversally averaged EELS signal in Fig. 2d–f. Evaluating equation (1) at the GSP-induced resonance energies produces the grey lines shown in Fig. 2d–f, which accurately captures the spatial distribution of the GSP-induced modes. Only for the case of *m*=4, that is, at the resonance energy 2.53 eV, do we observe a slight discrepancy in plasmon wavelength near the slot terminations. This can again be related to the only approximate relation in equation (4) at large energies, which also influences the calculation of the reflection coefficient. Besides this slight discrepancy, the analytical GSP model is impressively accurate and it is transparently describing both the spectral and spatial dependence of the EELS signal. The analytical GSP model also verifies that the experimentally observed resonances and EELS intensity maps are indeed consistent with the interpretation of counter-propagating slot GSPs producing standing-wave patterns in a Fabry–Perot manner. Thus, the resonances at 1.45, 2.03 and 2.53 eV along with the weak shoulder at ∼0.75 eV are strong experimental evidences for the broadband single-mode GSP guiding property of the ultranarrow slit, covering almost the entire visible and near-infrared spectral range.

The analytical GSP model provides an additional important point linked to the general interpretation of EELS, which we discuss briefly. Since the electron beam is modelled as a point source producing GSP waves, the EELS signal is then a measure of the efficiency of the electron beam to excite GSP modes in the resonators. Hence, a large EELS signal can be understood as a strong coupling between the electron beam and the GSP mode. This interpretation of EELS supplements the previously established understanding of EELS as related to the photonic local density of states^50^,^56^. However, this interpretation also implies that the EELS intensity maps, that is, spatial EELS profiles, cannot simply be understood as the plasmonic mode profile^32^,^33^. In particular for the slot GSP in this study, the main *x* and *y* electric-field components of the GSP have a different spatial dependence than the EELS intensity maps of Fig. 2c–f (Supplementary Fig. 1). Hence, the spatial EELS profiles should not be regarded as the GSP mode profiles, but rather as a map of the GSP excitation efficiency of the swift electrons during their interaction with a nontrivial superposition of the dispersive GSP modes.

### L-shaped slot

**Figure 3:**
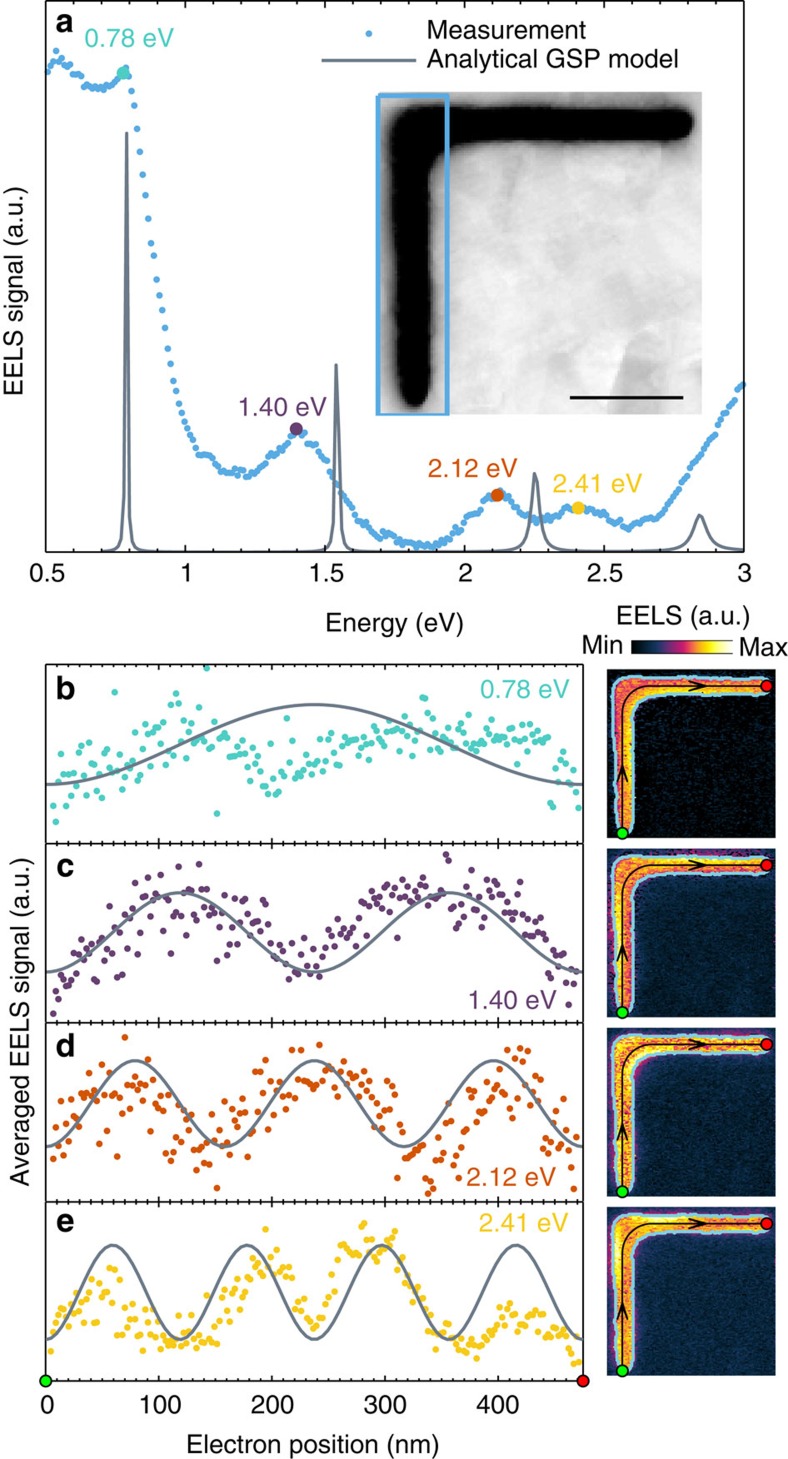
L-shaped slot resonator. (**a**) Total EELS signal from the enclosed blue box shown in the inset along with calculations using the analytical GSP model. Scale bar, 100 nm. (**b**–**e**) 1D EELS intensity profiles (left) at the GSP resonance energies 0.78, 1.40, 2.12 and 2.41 eV, respectively, along with calculations using the analytical GSP model. The profiles are determined by averaging the 2D EELS intensity maps (right) transversally to the GSP propagation direction (black line with arrows). Only EELS data in the air gap (enclosed light-blue box) is used for the averaging procedure.

One of the important requirements for a plasmonic mode to be suitable for nanoscale integrated circuits is a high transmission through sharp bends, that is, low back reflection, such that routing of light to desired locations can be achieved. We therefore characterize the bending property of the slot GSP mode by considering a slot resonator of similar length and width as the one in Fig. 2, but with a 90° bend in the middle of the resonator, see the inset of Fig. 3a for the STEM image. Given the symmetry of the L-shaped resonator in Fig. 3a, it is sufficient to consider only the EELS signal from one of the branches to map all of the modes. Figure 3a displays the EELS signal for the lower branch, where we observe four distinct resonances at energies 0.78, 1.40, 2.12 and 2.41 eV. All four energies are similar in value to the resonance energies observed for the straight slot resonator (Fig. 2), although the lowest-energy resonance was previously only observed as a weak shoulder. Hence, we anticipate these modes to be induced by counter-propagating slot GSPs with a similar propagation length *L* as for the case of the straight slot resonator, suggesting that the slot GSP bends around the 90° corner without significant back reflection or phase change. This interpretation is confirmed when examining the spatial distribution of the EELS signal, as shown in Fig. 3b–e. Once again, by averaging the EELS signal in the air gap transversally to the GSP propagation direction (see Methods for details), we extract one-dimensional EELS profiles, which clearly show the expected standing-wave pattern produced by the slot GSPs. For simplicity, we approximate the geometry of the waveguide bend by a 90° circular arc, whose radius can be regarded as the curvature of the bend. From inspection of the STEM image in the inset of Fig. 3a the radius of curvature is roughly 45 nm. The negligible back reflection from the corner seen in the L-shaped resonator of Fig. 3 has also been observed in other L-shaped resonators of similar total length (*L*≈500 nm), but with 90° bends positioned differently such that the branches have different lengths (data not included). In these cases, we also observe a very similar spectral and spatial EELS behaviour, confirming the high transmission of the slot GSP through sharp bends.

The low back reflection at the corner justifies the comparison of the experimental results of the L-shaped resonator with the analytical GSP model, which is based on a straight slot. Indeed, Fig. 3a displays accurate spectral agreement between the observed and calculated resonance energies, with the same decrease in accuracy of the theoretical model for larger energies (as a consequence of the validity of equation (4)). Comparison of spatial EELS profiles (Fig. 3b–e) also shows good agreement between theory and experiments, although we note that the EELS signal drops at the corner, which is more pronounced for the modes with standing-wave patterns that have maxima at the corner (that is, *m*=1 and *m*=3). In particular, the lowest-order mode (*m*=1) shows this effect in Fig. 3b. We observe the same phenomenon at T-shaped junctions, which we discuss later, and cross-shaped junctions (Supplementary Fig. 5). Ruling out FIB-induced silver modification at the corners (Supplementary Fig. 6), we interpret the decreased EELS signal to be a consequence of the decreased GSP excitation efficiency of the electron beam. We relate this effect to (i) the weaker charge distribution due to the lack of a direct opposing metal boundary (compared with the straight sections) and (ii) the decreased local GSP effective index (due to an increased width), providing less mode confinement and weaker electric field components. The increase in slot width also decreases the averaged EELS signal. Disregarding the corner-related decrease in EELS signal, we find once again excellent agreement between experiments and the analytical GSP model. More importantly, we have successfully showed the bending property of the slot GSP with negligible bending loss and high transmission.

### T-shaped slot

**Figure 4:**
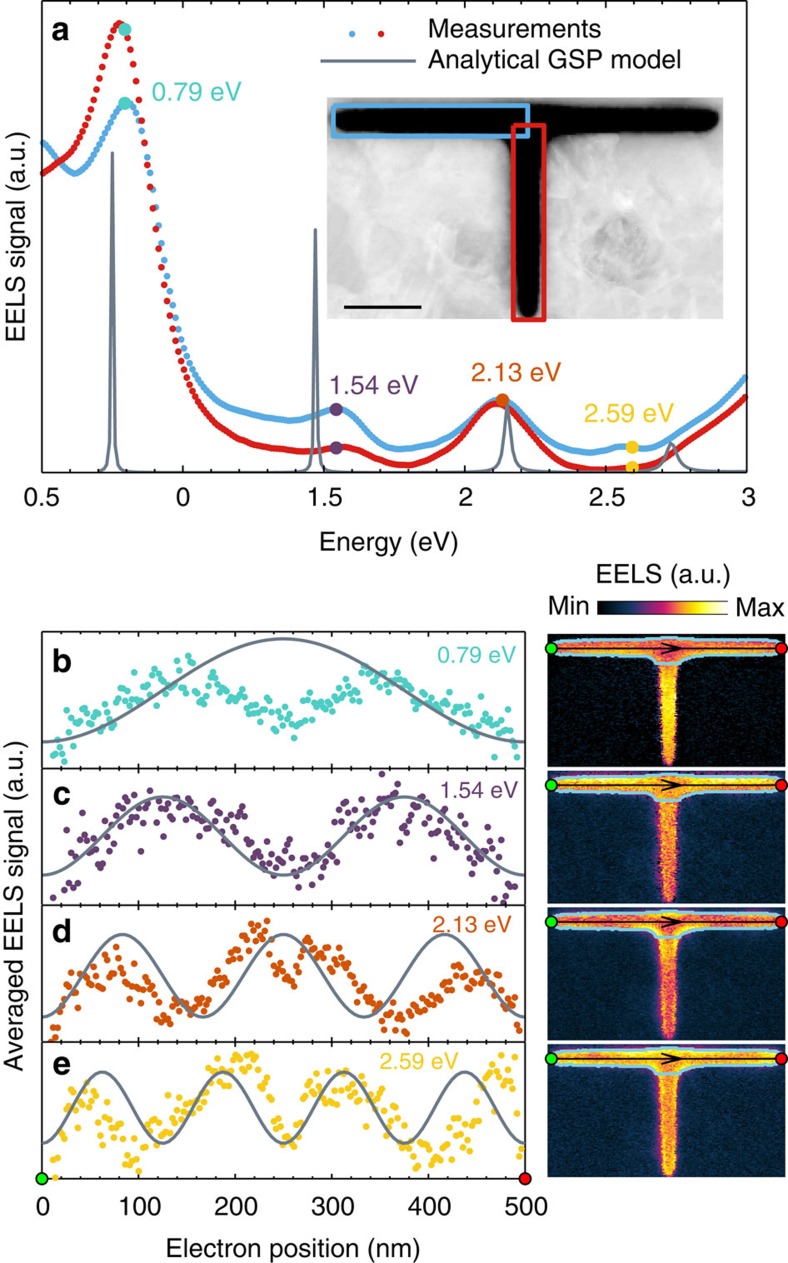
Equal-length T-shaped slot resonator. (**a**) Total EELS signal from the enclosed blue and red boxes shown in the inset along with calculations using the analytical GSP model. The lower (upper) branch is ∼250 nm (500 nm) in length. Scale bar, 100 nm. (**b**–**e**) 1D EELS intensity profiles (left) at the GSP resonance energies 0.79, 1.54, 2.13 and 2.59 eV, respectively, along with calculations using the analytical GSP model. The profiles are determined by averaging the 2D EELS intensity maps (right) transversally to the GSP propagation direction (black line with arrows). Only EELS data within the enclosed light-blue box is used for the averaging procedure.

Nanoscale on-chip modulation of light requires the interference or splitting of one or several beams at junctions in the waveguide circuit. Hence, understanding the behaviour of the slot GSP at junctions is essential for constructing a plasmonic circuit. We have therefore fabricated a T-shaped slot resonator, as shown in the inset of Fig. 4a, with upper and lower branch lengths of approximately 500 and 250 nm, respectively. The EELS signal from the upper and lower branches is shown in Fig. 4a in blue and red lines, respectively. Here we note the striking resemblance of the two EELS spectra. In particular, both branches show four GSP-induced resonances at the energies 0.79, 1.54, 2.13 and 2.59 eV. We also note that these resonance energies are similar to those observed in the L-shaped and straight slot resonators. The similarity in resonance energies of the two branches and the two other resonators are a consequence of the similar propagation length, leading to almost identical resonance conditions. A total length of ∼500 nm is traversed by the slot GSP regardless if the path is the upper branch only, or if the path, by splitting of the GSP, includes half of the upper branch and the lower branch. In other words, the two different paths give rise to degenerate GSP-induced resonances. The fact that no new GSP-induced resonances are observed, which reside only in the lower branch, is evidence for the splitting of the slot GSP at the T-shaped junction with minimal reflection. The transversally averaged spatial EELS profiles of the GSP-induced resonances in the upper branch are shown in Fig. 4b–e, which show the expected standing-wave patterns with increasing number of maxima (Supplementary Fig. 7 for the EELS profiles through the junction). Comparison with the analytical GSP model shows accurate spectral and spatial agreement, although the spatial profiles for the odd-numbered modes *m*=1 and *m*=3 differ at the junction. As discussed in relation to the L-shaped resonator, the experimentally observed dip in the EELS signal at the junction is a consequence of the decrease in GSP excitation efficiency of the electron, which is not accounted for in the analytical GSP model.

**Figure 5:**
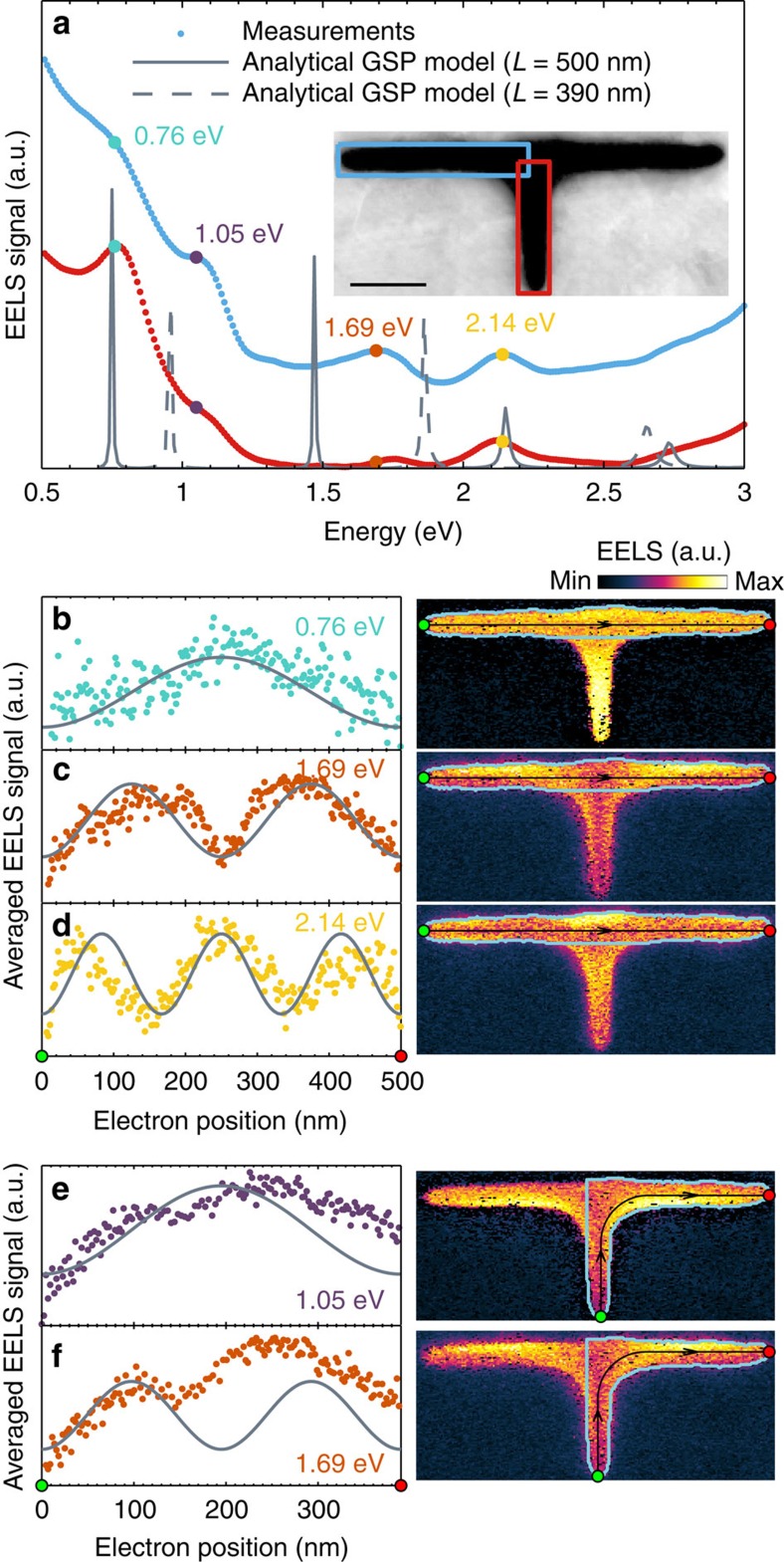
Unequal-length T-shaped slot resonator. (**a**) Total EELS signal from the enclosed blue and red boxes shown in the inset along with calculations using the analytical GSP model. The lower (upper) branch is ∼170 nm (500 nm) in length. Scale bar, 100 nm. (**b**–**d**) 1D EELS intensity profiles (left) at the GSP resonance energies 0.76, 1.69 and 2.14 eV, respectively, along with calculations using the analytical GSP model. The profiles are determined by averaging the 2D EELS intensity maps (right) transversally to the GSP propagation direction (black line with arrows). Only EELS data within the enclosed light-blue box is used for the averaging procedure. (**e**,**f**) Same as **b**–**d** at GSP resonance energies 1.05 and 1.69 eV, respectively, but transversally averaged along a shorter propagation path.

We can lift the degeneracy of the GSP-induced modes by changing the length of the lower branch in the T-shaped resonator, such that the two different paths have different lengths and thereby resonate at different energies. We consider this case in Fig. 5a, where we show the EELS signal from a T-shaped resonator with a lower branch of shorter length (∼170 nm). Thus, the path along the upper branch still has a length of ∼500 nm, while the GSP path involving the lower branch is now around 390 nm. From Fig. 5a, we note that this difference in path length leads to different EELS response from the two branches. In total, we observe four resonances at energies 0.76, 1.05, 1.69, and 2.14 eV. On the basis of the spatial profiles in Fig. 5b,d, the resonances at 0.76 and 2.14 eV are immediately identified as the *m*=1 and *m*=3 modes related to counter-propagating slot GSPs in the upper branch (that is, *L*≈500 nm). Similar resonance energies for these modes were also previously observed for the straight and L-shaped resonators. The interesting features are the two remaining resonances, which differ in energy from any of the previous slot GSP resonances. By considering the spatial profile in Fig. 5e and comparing with the analytical GSP model, we interpret the resonance at energy 1.05 eV as the first-order (*m*=1) GSP-induced mode associated with the shorter path (*L*≈390 nm). Surprisingly, for the resonance energy at 1.69 eV, we find that we can consider the spatial profile along both the long path (Fig. 5c) and the short path (Fig. 5f), showing in both cases two maxima. Interestingly, this suggests that the observed resonance is due to the hybridization of the second-order GSP modes (*m*=2) of the short and long paths. Summarizing, we have provided strong evidence for splitting of the slot GSP at junctions, which even leads to resonant excitations at other energies (compared with the straight and L-shaped resonators) due to change in path lengths.

## Discussion

**Figure 6:**
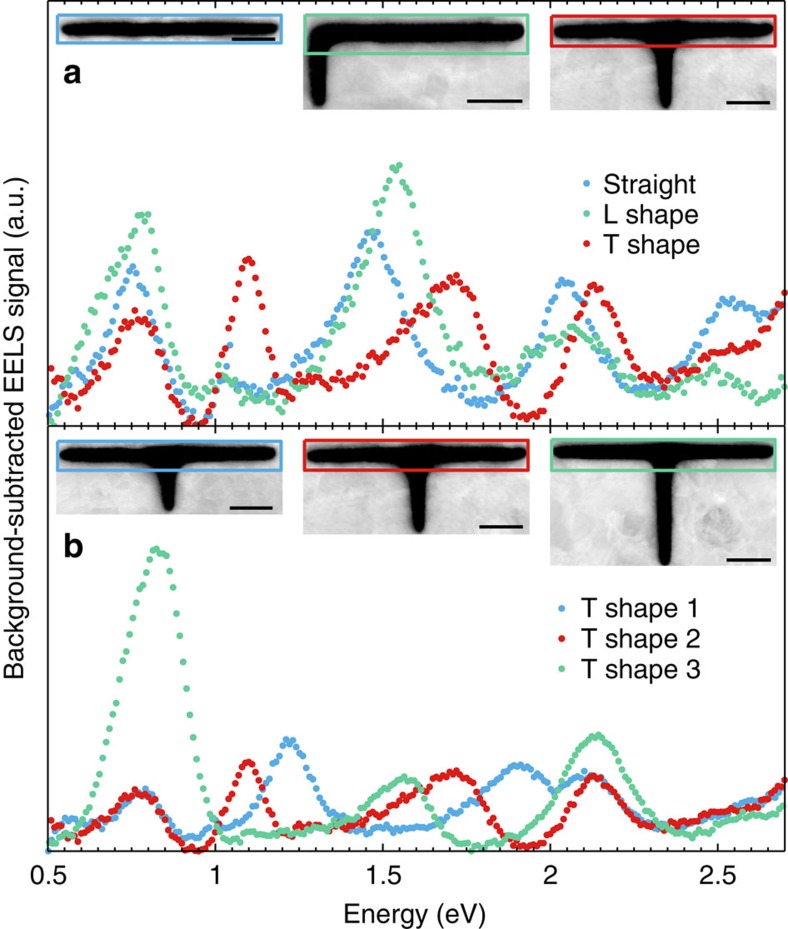
Comparison of slot resonators. (**a**,**b**) Background-subtracted EELS signal from the enclosed boxes shown in the insets. In **a**, the straight and L-shaped resonators show similar spectral response due to almost identical resonance conditions, while the T-shaped slot resonates at different energies due to the shorter lower branch. In **b**, the gradual change of length in the lower branch gives rise to significant variation in the GSP resonance energies, which can be monitored from the upper branch. Scale bar, 100 nm.

The properties of the slot GSP, including propagation, bending, and splitting, have been thoroughly investigated by EELS characterization of ultra-compact silver slot resonators. We have shown that the resonances in finite-length slots are primarily determined by the overall path length traversed by the slot GSP, regardless of bending around corners or splitting in junctions. In Fig. 6a, we emphasize this important point by comparing the EELS signal from straight, L-shaped, and T-shaped resonators. The L-shaped resonator has the corner situated ∼125 nm from the left slot termination (differing from the L-shaped resonator in Fig. 3), while the T-shaped resonator is the same as that in Fig. 5. Since the overall propagation distance of the slot GSP is ∼500 nm for the straight and the L-shaped slot resonators, the GSP-induced resonances occur at approximately the same energies. Hence, the slot GSP bends around the 90° corner effortlessly in a broad spectral range, making the GSP-induced resonant modes basically unaltered by the presence of the corner. In contrast, the introduction of a shorter path length in the T-shaped resonator (by splitting in the junction) gives rises to new resonances with, in this case, energies of 1.05 and 1.69 eV.

The dependence of the GSP-induced resonance energies on the lower branch length of the T-shaped resonator is investigated in more detail in Fig. 6b. Here we map the GSP-induced resonances by considering the EELS signal from the upper branch, which has a constant length of 500 nm in all three cases. The lower branch length increases from ∼125 nm through 170 to 250 nm. Here we clearly see that the first- and second-order (*m*=1 and *m*=2) modes related to the path length involving the lower branch decrease in resonance energy with increasing lower branch length (that is, overall path length). Hence, the GSP-induced resonances, as tracked from the upper branch, are extremely sensitive to the optical path length of the slot GSP. By straightforwardly converting the change in optical length of the lower branch into a change in refractive index while keeping the length fixed at 125 nm, we can estimate the sensitivity *S* of the T-shaped slot waveguide resonator, that is, the wavelength shift per refractive index unit (RIU). We find the refractive index sensitivities of the first-order and second-order modes to be *S*_*m*=1_≈550 nm/RIU and *S*_*m*=2_≈150 nm/RIU. Impressively, the sensitivity of the first-order mode is comparable to state-of-the-art plasmonic sensors of similar footprints^45^, while being ultra-compact with a volume of only ≈(0.5 × 0.15 × 0.15) μm^3^=0.01 μm^3^. Besides the sensitivity, another parameter for characterizing a sensor is the figure-of-merit relating to the resonance linewidth^45^. Unfortunately, accurate quantitative determination of plasmon linewidths with EELS is not readily possible due to the energy resolution of EELS, although progress in this direction has recently been achieved^57^,^58^. Since the EELS signal is connected to the photonic local density of states^56^,^59^, the marked changes in both the spatial and spectral EELS response of the upper branch can also be observed in the response of quantum emitters. Note that the GSP modes form the basis for the channel plasmon polaritons propagating along V-grooves, that were recently demonstrated to be well suitable for efficient coupling to individual quantum emitters^60^. Yet another perspective application of extremely confined GSP modes similar to those studied here can be their usage for resonant guided wave networks^61^,^62^.

Overall, extremely confined GSP modes studied in this work exhibit remarkable flexibility in their manipulation, a unique feature that, in our opinion, opens up new avenues for their exploitation in diverse areas of modern nanophotonics, ranging from ultra-compact resonators for refractive-index sensing and resonant guided-wave networks to quantum plasmonics.

## Methods

### Fabrication

Commercially available silicon nitride TEM membranes (10 nm thickness) are used as a thin planar substrate. A 150 nm thick smooth silver film is then deposited onto the substrate using e-beam evaporation. Subsequently, different slot geometries are milled into the silver film using focused ion beam (FIB) with a FEI Helios 600i dual FIB/SEM tool.

Due to short periods of air exposure of the samples, we could not effectively prevent the oxidation or sulfidation of silver at the top interface of the sample. However, the air-induced changes to the top interface primarily affects the surface plasmon excited at the corresponding interface and thus have no significant impact on the gap surface-plasmon modes.

### EELS measurements

The EELS measurements are performed with a FEI Titan transmission electron microscope equipped with a monochromator and an image corrector. The microscope is operated in STEM mode at an acceleration voltage of 300 kV, providing a spot size of ∼0.3 nm and an energy resolution of 0.10 eV (measured as the full-width at half-maximum of the zero-loss peak). The microscope is equipped with a Quantum 966 electron energy-loss spectrometer and the Gatan DigiScan acquisition system, which records an entire EELS intensity map in 20 to 40 min, depending on the number of pixels. A C3 aperture size of 50 μm, camera length of 38 mm and entrance aperture of 2.5 mm were used for the EELS measurements. This correspond to convergence and collection angles of 8.4 and 18.3 mrad, respectively. A spectral dispersion of 0.01 eV per pixel was used in all of the spectra collection. In addition, we utilize the automatic drift and dark-current correction function included in the acquisition system. The individual EELS spectrum of the EELS intensity maps (with pixel sizes typically of 2–2.5 nm) are recorded with acquisition times ranging from 10 to 12 ms.

The first post-processing step of the EELS spectra is the removal of the zero-loss peak using the reflected-tail method, where the negative energy part of the zero-loss peak is mirrored around the zero-energy point to reconstruct the zero-loss peak and subsequently subtracted from the spectra. The resonance energies are then extracted by fitting a Gaussian function using a nonlinear least-squares fit. All of the resonant EELS intensity maps shown in this paper depict the summed EELS signal in a 0.15 eV spectral window centred at the extracted resonance energies. In Fig. 6, we additionally subtract the background contribution to the EELS signal by fitting two linear functions; the first in the energy range 0.5 eV to around 1 eV and the second in the energy range from around 1 to 2.7 eV. Both linear fits are performed by manually excluding the data of the gap surface-plasmon peaks.

To compare the EELS intensity maps at resonance with the one-dimensional analytical GSP model, we convert the two-dimensional EELS maps into one-dimensional EELS line profiles using an averaging procedure. To increase the signal-to-noise ratio, we use only the EELS data in the air gap of the GSP resonators. Thus, we initially determine the boundary between the air gap and the surrounding silver using the Image Processing Toolbox in MATLAB. By converting the grey-scale STEM image into a binary image using the threshold determined from Otsu's method, we extract the air–silver boundary. Subsequently, we average the EELS data within the closed boundary transversely to the GSP propagation direction. For the straight slot in Fig. 2, this approach amounts to averaging the EELS data along the *y* direction. However, for the resonators with bends and splits (L- and T-shaped), we fit the corner with a 90° circular arc. Averaging the EELS data in a corner is then performed along straight lines perpendicular to the fitted arc (that is, in the radial direction). The radius of the fitted circular arc is also used as a measure for the radius of curvature of the fabricated bends.

### Simulations

The derivation of the one-dimensional analytical GSP model for calculating the EELS signal is presented in detail in Supplementary Note 1.

The EELS signal from the top and bottom surface plasmons (Fig. 2a) is calculated using a 2D model in COMSOL Multiphysics (version 5.1), where the electron beam is set to travel parallel to the metal-dielectric interfaces. The electron beam is modelled as an out-of-plane line current with wave number *k*_*e*_=*ω*/*v*, where *v* is the electron speed. Although the electron beam travels perpendicular to the metal-dielectric interfaces in the experiments, our only interest is in evaluating the EELS signal stemming from the excitation of surface plasmons at their non-retarded frequencies. This can be achieved using the 2D implementation by setting a low electron velocity (here, *v*=0.5*c*) and positioning the electron beam to always travel in the vacuum part of the domain (a distance of 5 nm to the outer interface is chosen). In this manner, the main contribution to the theoretical EELS signal will be from the excitation of surface plasmons at their non-retarded energies, just as in the experiments. In all calculations, we use the permittivity for silver from ref. 55, while the permittivity for silicon nitride is from ref. 63. A thickness of 10 nm is used to model the silicon nitride layer from the TEM membrane.

### Data availability

Data available on request from the authors.

## Additional information

**How to cite this article:** Raza, S. *et al*. Electron energy-loss spectroscopy of branched gap plasmon resonators. *Nat. Commun.* **7,** 13790 doi: 10.1038/ncomms13790 (2016).

**Publisher's note:** Springer Nature remains neutral with regard to jurisdictional claims in published maps and institutional affiliations.

## Supplementary Material

Supplementary Information Supplementary Figures, Supplementary Note and Supplementary References
