# Supplementary material for: Electron energy-loss spectroscopy of branched gap plasmon resonators
Source: Nat Commun. 2016 Dec 16;7:13790. doi: 10.1038/ncomms13790 (PMC5171719; doi:10.1038/ncomms13790)
Supplement: Supplementary Information — Supplementary Figures, Supplementary Note and Supplementary References [file ncomms13790-s1.pdf]

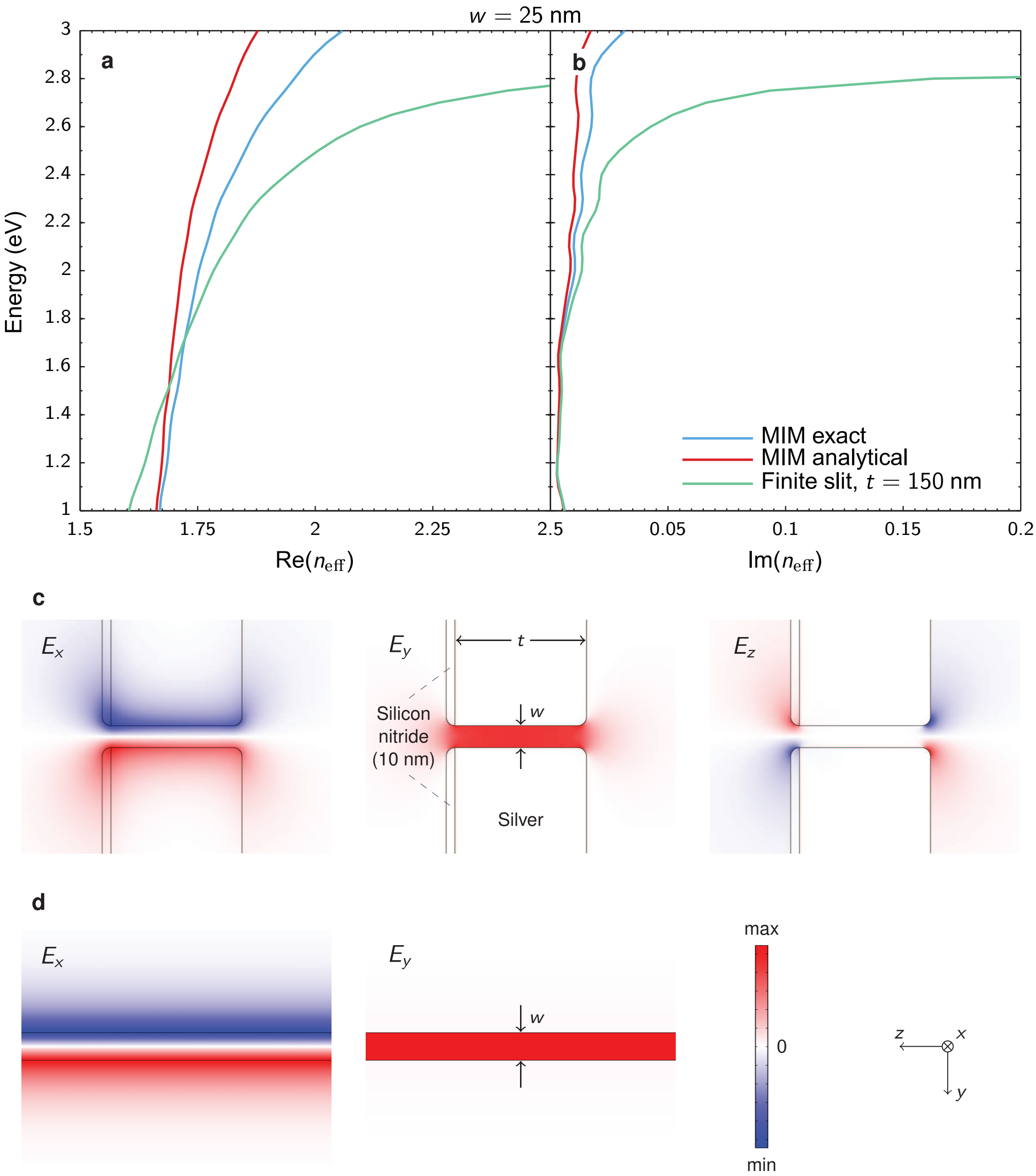

Supplementary Figure 1: Effective index and mode profiles. (**a,b**) Gap surface-plasmon mode dispersions of the MIM geometry by solving the exact transcendental equation (blue lines) and by using the analytical approximate relation (red lines). The dispersion relation for a finite silver slot of thickness $t = 150$ nm with a 10 nm layer of silicon nitride has been solved numerically and is also shown (green lines). For the finite slot, all corners have been rounded with a curvature of 10 nm. In all cases, the width is $w = 25$ nm. **(c,d)** Electric field components of the gap surface-plasmon modes of the finite slot and MIM geometries, respectively, at the energy $E = 2$ eV.

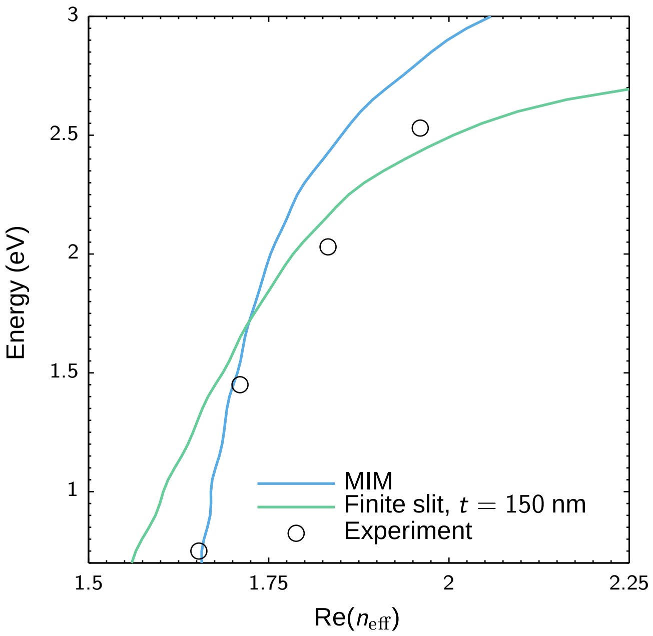

Supplementary Figure 2: Experimental effective mode index. Gap-surface plasmon dispersion relations determined numerically (as in Supplementary Fig. 4) and experimentally from the standing-wave patterns of the straight slot resonator (Figure 2).[2–5]

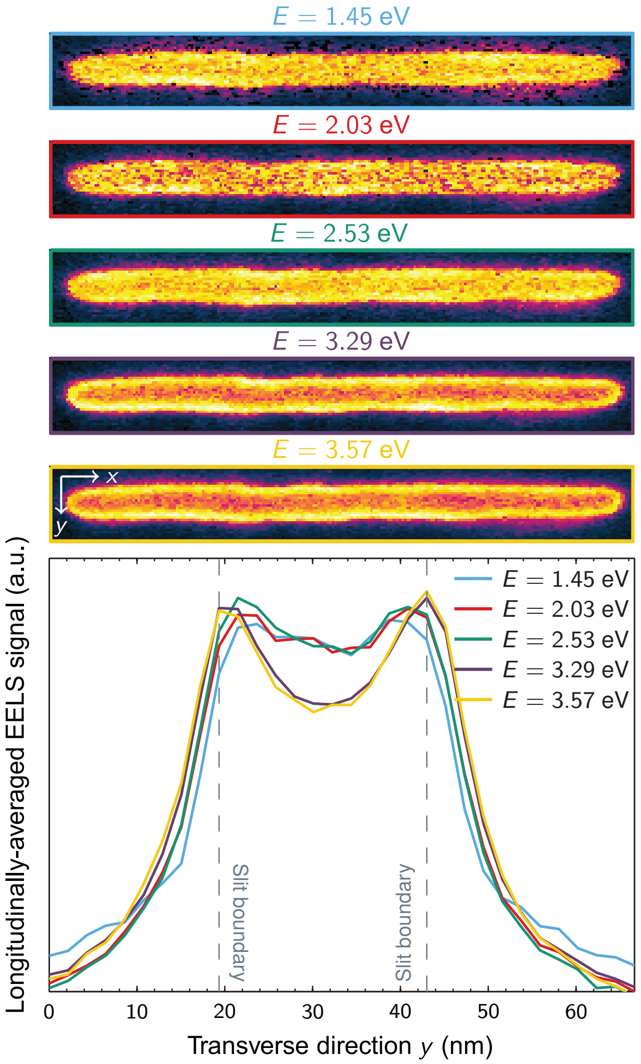

Supplementary Figure 3: Longitudinal EELS averaging. 1D averaged EELS intensity profiles (bottom) at the resonance energies of the EELS data from Figure 2b in the main manuscript. The profiles are determined by averaging the 2D EELS intensity maps (top) longitudinal to the GSP propagation direction, i.e., in the x-direction. For comparison, all spectra have unity area. The three lowest-energy resonances correspond to excitation of GSP modes with increasing order, while the two higher-energy resonances are due to excitations of SP modes at the bottom silicon nitride and top air interfaces, respectively. Interestingly, the SP modes are most efficiently excited exactly at the boundary between the air gap and the silver as expected from theory,[1] while the GSP modes have their maximum excitation efficiency several nanometers inside the air gap.

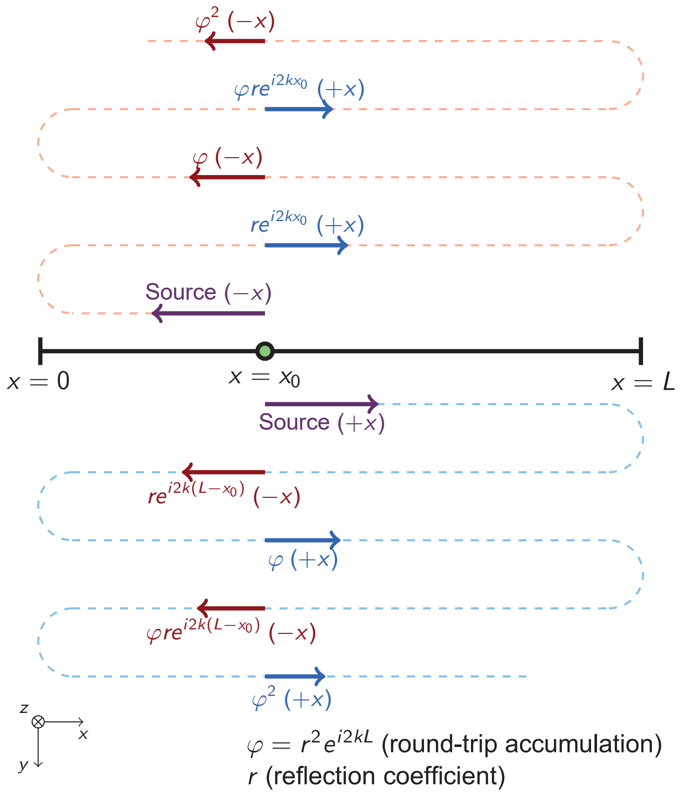

Supplementary Figure 4: Analytical GSP model. Schematic illustration of one-dimensional slot which shows the propagation and reflection of gap plasmon modes induced by the electron (source) positioned at x_0.

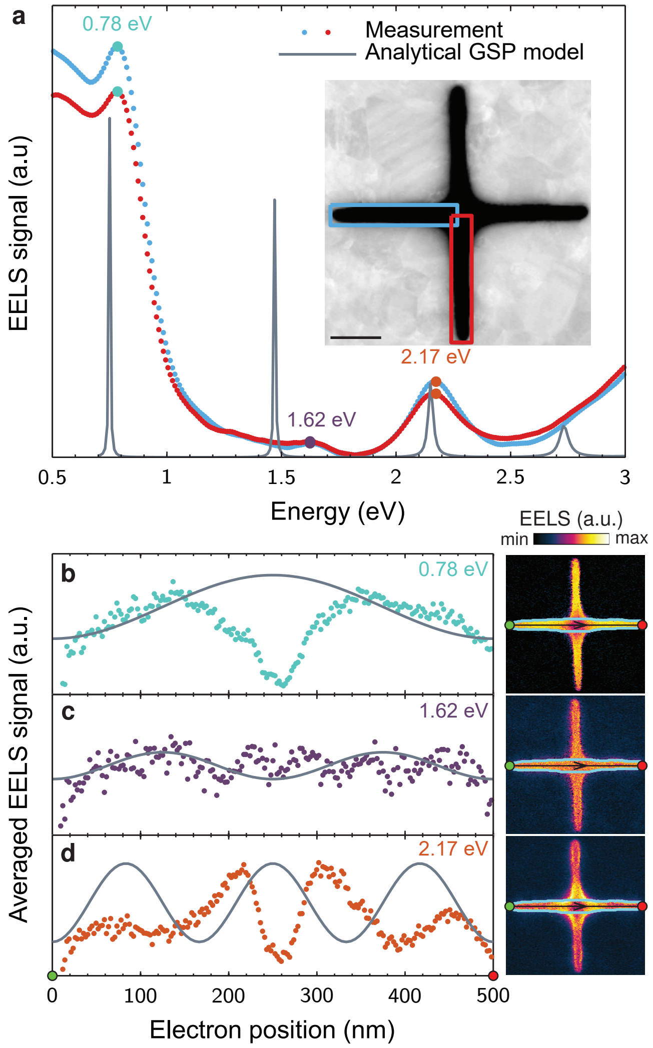

Supplementary Figure 5: Equal-length cross-shaped slot resonator. (**a**) Total EELS signal from the enclosed blue and red boxes shown in the inset along with calculations using the analytical GSP model. Each branch is approximately 250 nm in length. Scale bar is 100 nm. (**b-d**) 1D averaged EELS intensity profiles (left) at the GSP resonance energies 0.78 eV, 1.62 eV, and 2.17 eV, respectively, along with calculations using the analytical GSP model. The profiles are determined by averaging the 2D EELS intensity maps (right) transverse to the GSP propagation direction (black line with arrow). Only EELS data within the enclosed light blue box is used for the averaging procedure. A clear dip in the averaged EELS signal is observed at the intersection due to decreased GSP excitation efficiency.

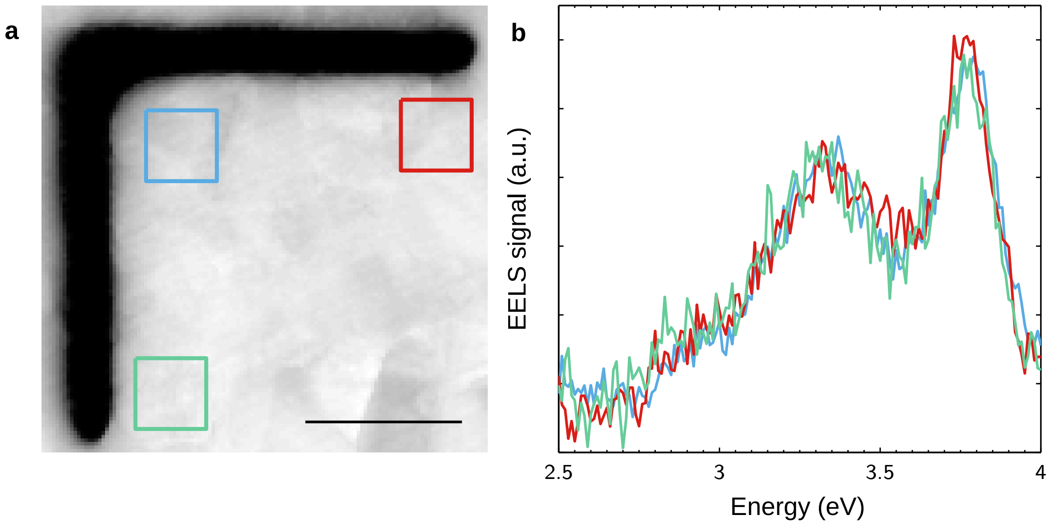

Supplementary Figure 6: EELS signal at the corner region. (**a,b**) Comparison of the EELS signal from the corner region (blue line) with two regions close to the slot terminations (red and green lines). The observed EELS peaks are due to excitations of the surface plasmons at the top and bottom interfaces of the sample (as explained in the main text). The similarity in the observed EELS signal of the surface plasmon excitations provides strong evidence that the silver film is not significantly altered at the corner compared to the terminations. Scale bar is 100 nm.

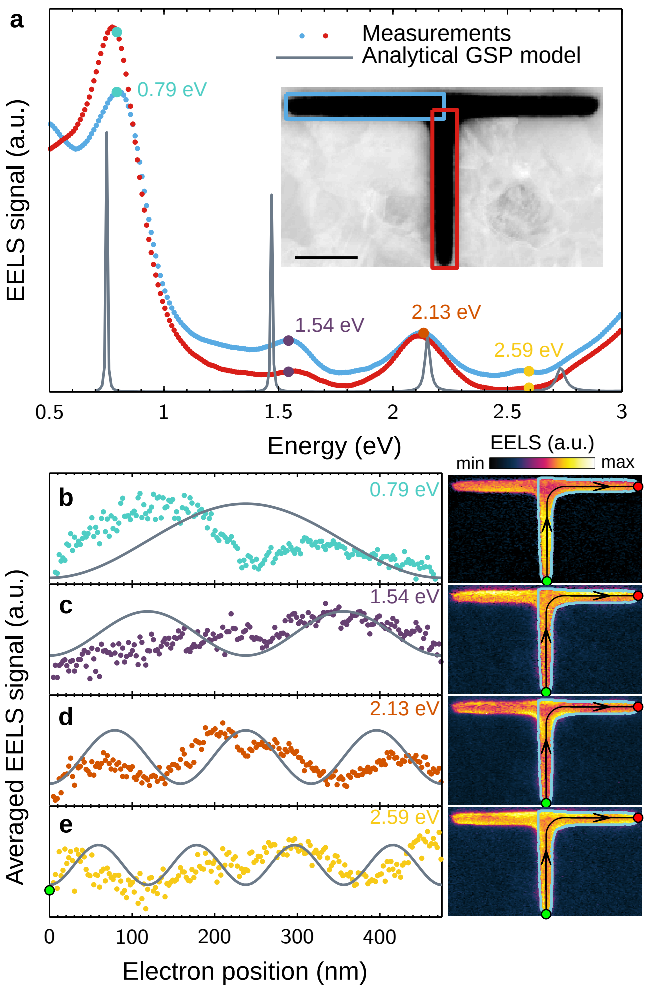

Supplementary Figure 7: Equal-length T-shaped resonator. (**a-e**) Similar to Figure 4 of the main manuscript but now with the EELS signal averaged along the pathway through the junction. The 1D EELS profiles along the bend differ from the profiles along the upper branch (shown in Figure 4). We attribute these changes to different pathways, which produce resonances at the same energy (degenerate modes). The decrease in the EELS signal in the upper branch of the $m = 1$ mode comes from the slight phase shift between the pathway involving only the upper branch and that involving the lower branch. The $m = 2$ and $m = 4$ modes are distorted in the lower branch due to interference with the resonant modes of the lower branch (which is half the length of the other pathways). Scale bar is 100 nm.

Supplementary Note 1: Analytical gap surface-plasmon model

In this Note, we present a detailed derivation of the one-dimensional analytical gap surface-plasmon (GSP) model for determining the EELS signal, which has been utilized in the main manuscript to interpret our experimental observations. We begin by briefly discussing the classical approach for calculating the EELS signal $\Gamma(\omega)$, which is given by the general relation[1]

$$\Gamma(\omega) = \frac{e}{\pi\hbar\omega v}\int \mathrm{d}z \mathrm{Re}\left[e^{-ik_\mathrm{e}z}\mathbf{v}\cdot\mathbf{E}^{\mathrm{ind}}(x_0,y_0,z,\omega)\right]. \tag{1}$$

Here, $\mathbf{v} = v\hat{\mathbf{z}}$ is the velocity vector and defines the direction of the straight-line path of the electron beam, while (x_0, y_0) are the coordinates of the electron beam impact in the plane normal to the propagation direction. Finally, $k_\mathrm{e} = \omega/v$ denotes the electron wave vector and $\mathbf{E}^{\mathrm{ind}}$ is the induced electric field, i.e., the total field subtracted the source field of the electron. Physically, Eq. (1) relates the probability of an energy loss event to the work performed by the induced electric field along the trajectory of the electron beam.

Since the structures studied experimentally are only $t = 150$ nm thick in the z-direction, we make two approximations: (1) we perform the Taylor expansion $e^{-ik_\mathrm{e}z} \simeq 1$, and (2) we assume the z-component of the electric field to be weakly dependent on the z-coordinate. We then perform the integral in Eq. (1) over the length of the structure t

$$\Gamma(\omega) \simeq \frac{et}{\pi\hbar\omega}\mathrm{Re}\left[E_z^{\mathrm{ind}}(x_0,y_0,\omega)\right], \tag{2}$$

which is the approximate relation we use for evaluating the EELS signal. The task now is to determine the induced electric field.

To this end, we consider a one-dimensional slot of length L, which supports GSP modes with the effective index $n_{\mathrm{eff}}(w)$ that depends on the slot width w. We set the coordinate system such that the slot is positioned along the x-axis in the region $0 < x < L$, see also the schematic in Supplementary Fig. 4. For $x < 0$ and $x > L$, the material is silver. The electron beam is assumed to act as a point source that induces forward- ($+x$) and backward-propagating ($-x$) GSP waves.

We point out that we are interested in determining the z-component of the induced electric field, which is present only because the structure has a finite thickness (i.e., $t = 150$ nm). An infinitely thick slot would correspond to the metal-insulator-metal (MIM) case, where the supported GSP mode only has x and y electric field components. However, in our case, we assume that the z-component of the electric field behaves similar to the two other components and can therefore be described by GSP waves close in nature to those in the MIM geometry. Positioning the electron beam at x_0, the forward- and backward-propagating GSP waves are described by the plane waves (assuming an $e^{-i\omega t}$ time dependence)

$$\begin{aligned} u_\mathrm{f}(x) &= E_0 e^{ik(x-x_0)}, \\ u_\mathrm{b}(x) &= E_0 e^{-ik(x-x_0)}, \end{aligned} \tag{3}$$

respectively, where $k = k_0 n_{\mathrm{eff}}$ is the GSP wave vector, $k_0 = \omega/c$ is the vacuum wave vector, and E_0 is the wave amplitude. To calculate the induced wave $u(x)$ in the slot, we must track the propagation and reflections of the forward- and backward-moving GSP waves. We sum up each

reflected contribution at the position of the electron x_0. Referring to Supplementary Fig. 4, we see that the waves traveling in the forward ($+x$) direction at the position x_0 have two contributions: (1) from the direct round trips of the initial forward-propagating wave (shown as blue arrows in the lower part of Supplementary Fig. 4), and (2) from the round trips of the backward-propagating wave, which has undergone a single reflection and thereby moving the in the $+x$ direction (shown as blue arrows in the upper part of Supplementary Fig. 4). The first contribution amounts to the following sum

$$u_{\text{f}}^{(1)}(x) = E_0 \varphi e^{ik(x-x_0)} \left[1 + \varphi + \varphi^2 + \varphi^3 + \cdots\right] = E_0 \frac{\varphi}{1-\varphi} e^{ik(x-x_0)}, \tag{4}$$

where r denotes the reflection coefficient and $\varphi = r^2 e^{i2kL}$ is the round-trip phase and amplitude accumulation. In the last equality of Eq. (4), we utilize the convergence of a geometric series with $|\varphi| < 1$. The second contribution can be written as the following sum

$$u_{\text{f}}^{(2)}(x) = E_0 r e^{i2kx_0} e^{ik(x-x_0)} \left[1 + \varphi + \varphi^2 + \varphi^3 + \cdots\right] = E_0 \frac{r e^{i2kx_0}}{1-\varphi} e^{ik(x-x_0)}. \tag{5}$$

The term re^{i2kx_0} is due to the propagation and reflection of the initially backward-propagating wave. The total forward-propagating wave is the sum of Eqs. (4) and (5)

$$u_{\text{f}}^{\text{tot}}(x) = u_{\text{f}}^{(1)}(x) + u_{\text{f}}^{(2)}(x) = E_0 \frac{\varphi + r e^{i2kx_0}}{1-\varphi} e^{ik(x-x_0)}. \tag{6}$$

Applying the same line of thought, we sum up the two contributions propagating in the $-x$ direction and find

$$u_{\text{b}}^{\text{tot}}(x) = E_0 \frac{\varphi + r e^{i2k(L-x_0)}}{1-\varphi} e^{-ik(x-x_0)}. \tag{7}$$

The total field in the slot $u(x)$ induced by the electron source is then the sum of Eqs. (6) and (7)

$$u(x) = E_0 \frac{1}{1-\varphi} \left\{ \left[\varphi + r e^{i2kx_0}\right] e^{ik(x-x_0)} + \left[\varphi + r e^{i2k(L-x_0)}\right] e^{-ik(x-x_0)} \right\} \tag{8}$$

From Eq. (8), we note that the resonance condition is given by the relation $r^2 e^{i2kL} = 1$ which can be rewritten to the relation $2kL + 2\arg(r) = 2m\pi$, where $\arg(r)$ is the phase accumulation upon reflection and m is a positive integer. In other words, resonance occurs when the accumulated round-trip phase equals a non-negative integer value of 2π.

The EELS signal $\Gamma(x_0, \omega)$ is proportional to the real part of the induced field at the position of the electron x_0 [see Eq. (2)], i.e.,

$$\Gamma(x_0, \omega) \propto \text{Re}\left[u(x = x_0)\right] = \text{Re}\left\{ 2E_0 \frac{r e^{ikL}}{1 - r^2 e^{i2kL}} \left\{ r e^{ikL} + \cos\left[k(L - 2x_0)\right] \right\} \right\}. \tag{9}$$

Equation (9) constitutes our main result and is applied for calculating the EELS signal. Since the only term that depends on the electron position x_0 is $\cos[k(w - 2x_0)]$, we anticipate a harmonic pattern in the resonant EELS line profiles, which is indeed also observed in the experimental data.

To evaluate Eq. (9) we need expressions for the reflection coefficient r and the GSP wave vector k. The reflection coefficient is approximated with the Fresnel equation for a normal-incidence plane wave

$$r \simeq \frac{n_{\text{eff}} - n_{\text{Ag}}}{n_{\text{eff}} + n_{\text{Ag}}}, \tag{10}$$

where $n_{\text{Ag}} = \sqrt{\varepsilon_{\text{Ag}}}$ is the complex refractive index for silver.[6] We are now left with the task of calculating the GSP wave vector and thereby also the effective index. To this end we note that the slot width of the resonators studied in this work is $w \approx 25$ nm, which is significantly smaller than the thickness of the silver layer ($t \approx 150$ nm). Thus, we approximate the mode in the finite-thickness slot with the MIM GSP mode (i.e., infinitely thick slit), which has the dispersion relation described by the transcendental equation[7,8]

$$\tanh\left(\frac{\sqrt{k^2 - k_0^2\varepsilon_{\text{d}}}\,w}{2}\right) = -\frac{\varepsilon_{\text{d}}\sqrt{k^2 - k_0^2\varepsilon_{\text{Ag}}}}{\varepsilon_{\text{Ag}}\sqrt{k^2 - k_0^2\varepsilon_{\text{d}}}}, \tag{11}$$

where ε_{d} is the permittivity of the sandwiched dielectric layer, which in our case is air, i.e., $\varepsilon_{\text{d}} = 1$. Instead of numerically solving Eq. (11) for the exact GSP wave vector, we realize that we can use the approximate analytical relation[8]

$$k \simeq k_0\sqrt{1 - \frac{2\sqrt{1-\varepsilon_{\text{Ag}}}}{\varepsilon_{\text{Ag}}k_0 w}}, \tag{12}$$

where we have inserted $\varepsilon_{\text{d}} = 1$ and which is valid for $w > 2/(k_0|\varepsilon_{\text{Ag}}|)$, appropriate for our structures. Hence, we are now able to calculate the EELS signal in Eq. (9) analytically.

To verify that using the approximate relation in Eq. (12) is justified, we study in detail the GSP dispersion relations and their corresponding electric field components in Supplementary Fig. 1. In particular, we compare the approximate relation of Eq. (12) with the exact MIM solution [Eq. (11)] and with the GSP mode of a finite silver slot ($t = 150$ nm) with a 10 nm layer of silicon nitride[9] (modelling the TEM membrane). The latter is calculated numerically and constitutes the most accurate theoretical representation of the GSP mode excited experimentally. For the comparison we choose the width to be $w = 25$ nm to match the fabricated structures. From Supplementary Fig. 1a,b, we see that the approximate MIM relation accurately matches the dispersion relation of the finite slot for low energies. In fact, we find that up to an energy of 2.5 eV, there is only a 12% relative error between the effective index calculated using the approximate relation and the index of the finite slot. Additionally, Supplementary Fig. 1c,d reveals that the longitudinal E_x and transverse E_y components of the finite slot and the infinite MIM geometry are quite similar, explaining the comparable magnitude of the effective indices. As the energy increases, the electric field components of the finite slot GSP mode deviate in nature from the MIM GSP mode by localizing near the silver-silicon nitride interface, which drastically increases the effective index. Hence, for energies above approximately 2.5 eV the approximate relation becomes inaccurate. Fortunately, most of the experimentally observed resonances occur below this energy threshold, justifying the use of the approximate analytical MIM relation to calculate the effective index of GSP mode.

Supplementary References

1. García de Abajo, F. J. Optical excitations in electron microscopy. *Rev. Mod. Phys.* **82**, 209–275 (2010).

2. Nicoletti, O. *et al.* Surface plasmon modes of a single silver nanorod: an electron energy loss study. *Opt. Express* **19**, 15371–15379 (2011).

3. Rossouw, D., Couillard, M., Vickery, J., Kumacheva, E. & Botton, G. A. Multipolar plasmonic resonances in silver nanowire antennas imaged with a subnanometer electron probe. *Nano Lett.* **11**, 1499–1504 (2011).

4. Rossouw, D. & Botton, G. A. Plasmonic response of bent silver nanowires for nanophotonic subwavelength waveguiding. *Phys. Rev. Lett.* **110**, 066801 (2013).

5. Schoen, D. T., Atre, A. C., García-Etxarri, A., Dionne, J. A. & Brongersma, M. L. Probing complex reflection coefficients in one-dimensional surface plasmon polariton waveguides and cavities using STEM EELS. *Nano Lett.* **15**, 120–126 (2015).

6. Johnson, P. B. & Christy, R. W. Optical constants of the noble metals. *Phys. Rev. B* **6**, 4370–4379 (1972).

7. Economou, E. N. Surface plasmons in thin films. *Phys. Rev.* **182**, 539–554 (1969).

8. Bozhevolnyi, S. I. & Jung, J. Scaling for gap plasmon based waveguides. *Opt. Express* **16**, 2676–2684 (2008).

9. Philipp, H. R. Optical properties of silicon nitride. *J. Electrochem. Soc.* **120**, 295–300 (1973).
